# Fasting enhances extinction retention and prevents the return of fear in humans

**DOI:** 10.1038/s41398-018-0260-1

**Published:** 2018-10-09

**Authors:** Le Shi, Jiahui Deng, Sijing Chen, Jianyu Que, Yekun Sun, Zhong Wang, Xiaojie Guo, Ying Han, Yuxin Zhou, Xiujun Zhang, Wen Xie, Xiao Lin, Jie Shi, Lin Lu

**Affiliations:** 1 Department of Pharmacology, School of Basic Medical Sciences, Peking University Health Science Center, Beijing, 100191 China; 2 National Institute on Drug Dependence and Beijing Key Laboratory of Drug Dependence, Peking University, Beijing, 100191 China; 3 Peking University Sixth Hospital, Peking University Institute of Mental Health Key Laboratory of Mental Health, Ministry of Health (Peking University), National Clinical Research Center for Mental Disorders (Peking University Sixth Hospital), Beijing, 100191 China; 4 Institute of Psychology, North China University of Science and Technology, Tangshan, 063009 China; 5 Department of Medical Psychology, Anhui Medical University, Hefei, 230032 China; 6 Department of Neuroscience, Allegheny College, Meadville, 16335 USA; 7 Mental Health Center of Anhui Province, Hefei, 230032 China; 8 Peking-Tsinghua Center for Life Sciences and PKU-IDG/McGovern Institute for Brain Research, Peking University, Beijing, 100191 China

## Abstract

Fear is prone to return following extinction that is the basis of exposure therapy for fear-related disorders. Manipulations that enhance the extinction process can be beneficial for treatment. Animal studies have shown that fasting or caloric restriction can enhance extinction and inhibit the return of fear. The present study examined the effects of fasting on fear acquisition, extinction, and the return of fear in humans. One hundred and twenty-five male participants were randomized into a fasting group and food group and exposed to a Pavlovian fear conditioning paradigm. Changes in plasma cortisol and ghrelin levels were examined using enzyme-linked immunosorbent assays. One-night fasting had no effect on fear acquisition but enhanced fear extinction retention and prevented the return of fear, and this effect persisted for at least 6 months. This procedure was also effective for remote fear memory. Plasma ghrelin levels were elevated after fasting and had a negative relationship with the fear response in spontaneous recovery test. However, overnight fasting did not affect cortisol levels. These findings indicate that fasting enhances extinction retention and prevents the return of fear, without influencing fear memory formation. We propose that this novel procedure may open new avenues for promoting extinction-based therapies for fear-related disorders.

## Introduction

Emotional memory is vital to the survival and development of individuals. However, excessive fear and anxiety can cause fear-related disorders, such as posttraumatic stress disorder^1^. The core feature of fear-related disorders is an excessive fear response to traumatic reminders. Currently, patients with fear-related disorders are often treated with extinction-based exposure therapy, in which the fear response is extinguished using repeated exposure to the original conditioned stimulus (CS) without pairing with the noxious unconditioned stimulus (US)^2^. Fear memory may be erased under certain circumstances^3–5^, but extinction training alone does not erase the original fear memory, and the decreased fear response often returns under some conditions, such as reinstatement^6^, renewal^7^, or spontaneous recovery^8^. This suggests that extinction training forms a new extinction memory that competes with the fear memory. Enhancing extinction memory may be helpful for achieving better treatment outcomes with extinction-based behavioral interventions.

Pharmacological agents can enhance extinction-based exposure therapy and prevent the return of fear^9–11^. However, most drugs that are used to promote extinction process cannot be translated from animal models to human studies because of concerns about safety, side effects, and the intracranial route of administration. This limits the clinical implications of pharmacological treatment. Recent studies indicated that device-based interventions, such as vagus nerve stimulation^12^, transcranial direct current stimulation^13^, and repeated transcranial magnetic stimulation^14^, facilitate the extinction process. In clinical practice, however, these methods are not feasible for widespread application because the equipment is relatively expensive, and some patients are intolerant to adverse reactions. Techniques that are effective, safe, and easy to implement and promote fear extinction memory are beneficial for the treatment of fear-related disorders.

Food restriction has been considered a useful method for improving memory performance by regulating hippocampal function^15,16^. The activation of emotion-related brain regions, such as the amygdala, orbitofrontal cortex, and hippocampus, was shown to be involved in the hunger-induced enhancement of memory in humans^15–19^, suggesting that fasting may affect extinction process. Acute fasting increases the persistence of memory across species^20,21^. Two studies reported that fasting for 16 h promoted fear extinction in rodents^21,22^. Short-term fasting prior to fear acquisition specifically impaired long-term fear memory, but fasting prior to fear extinction facilitated extinction learning^22^. Huang et al.^21^ suggested that acute fasting in mice enhanced fear extinction by activating ghrelin signaling in the amygdala. In humans, fasting may impact brain activation during working memory tasks and performance in several cognitive domains^23,24^. The present study investigated the effects of short-term fasting on fear acquisition, extinction, and the return of fear and developed an enhanced extinction retention training interference procedure to eliminate fear responses in humans.

## Materials and methods

### Participants

We recruited 125 young healthy native Han Chinese men through posters and online advertisements and conducted four experiments: Experiment 1 (food group: *n* = 17; fasting group: *n* = 18), Experiment 2 (food group: *n* = 21; fasting group: *n* = 24), Experiment 3 (food group: *n* = 13; fasting group: *n* = 17), Experiment 4 (food group: *n* = 22; fasting group: *n* = 23). The inclusion criteria were the following: (1) participants between 18 and 30 years of age with a body mass index (BMI) of 18.0–30.0 kg/m^2^, (2) generally good health as determined by a physician, and (3) the habit of eating breakfast every day and eating three meals per day regularly. The exclusion criteria included a history of metabolic, cardiovascular, neurological, or psychiatric disorders, substances abuse (alcohol, drugs, smoking, and medication), and physical impairment. Each participant was scheduled for a screening interview, during which they read the study protocol and signed a consent form that was approved by the Institutional Review Board of Peking University Sixth Hospital. The participants were paid USD $50 each. Before fear conditioning, the participants completed questionnaires that assessed their basic demographic characteristics (age, education, height, weight, and BMI), the Self-rating Depression Scale (SDS), the Self-rating Anxiety Scale (SAS), the Montreal Cognitive Assessment (MoCA), and the digit span test. The SDS and SAS were used to measure depression and anxiety, and the MoCA and digit span test were used to evaluate baseline cognitive function.

### Behavioral paradigm

Four behavioral experiments were conducted. Experiment 1 comprised fear conditioning and a fear memory test. Experiments 2–4 comprised fear conditioning, fear extinction, a spontaneous recovery test, and a reinstatement test. The protocol was based on previous studies^25–27^. For fear conditioning, the CS+ was paired with an electric shock (US) under a partial reinforcement schedule (50% reinforced). Another CS (CS−) was never paired with the US. The CSs were two colored squares (red or yellow). Two different orders of presentation were used to counterbalance designations of squares as the CS+ or CS−. The US was a shock to the wrist. Before the experiment, the US intensity was individually adjusted to reach maximum tolerable pain. Acquisition consisted of eight nonreinforced presentations of each CS, intermixed with eight additional reinforced CS+ presentations. To assess expectation of the reinforcer and avoid the influence of the electric shock on the skin conductance response (SCR), only nonreinforced trials of the CS+ were included when calculating the acquired fear response. In Experiment 1, the fear memory test was conducted 24 h after acquisition. The fear memory test had three trials, including three CS− presentations and three nonreinforced CS+ presentations. Fear expression was calculated as the average SCR of the three trials. Extinction training consisted of 15 CS+ presentations without electric shock and 15 CS− presentations. Extinction training was divided into three blocks with five CS+ and five CS− presentations each (blocks 1–3). The extinction score was calculated as the average of five CS+ and five CS− presentations in each block. No time interval, rest period, or signaled transitions occurred between blocks. Spontaneous recovery was tested 24 h after the end of extinction. To ensure that fear responses returned to baseline before reinstatement, there are 20 trials each containing one CS− presentation and one nonreinforced CS+ presentation in the spontaneous recovery test. The spontaneous recovery test score was calculated as the average SCR of the first three trials. One minute after the spontaneous recovery test, the participants received three unsignaled US presentations. The reinstatement test was followed by the unsignaled US, with no time gap. Three trials were conducted, including three CS− presentations and three nonreinforced CS+ presentations in the reinstatement test. The reinstatement test score was calculated as the average fear response of three trials. In all phases, the CSs were presented for 4 s, followed by an interstimulus interval of 8–12 s, during which the participants looked at a fixation point.

### Experimental design

In Experiment 1, we first investigated the effect of fasting on the acquisition of conditioned fear. Thirty-five participants (food group: *n* = 17; fasting group: *n* = 18) were recruited. On day 1, all of the participants were asked to eat breakfast, and they arrived at the laboratory at 8:00 AM. They were randomly assigned to one of two groups using random numbers. Both groups provided basic demographic information (age, education, height, weight, and BMI), completed the SAS, SDS, and MoCA, and performed the digit span test. The participants in the fasting group fasted for 16 h beginning at 6:00 PM on day 1. Fasting consisted of not consuming food or beverages that containing carbohydrates, fat, protein, alcohol, and so on, but they could drink water ad libitum. To guarantee that the participants complied with the instructions, we asked them to remain in the laboratory until noon the next day. On day 2, the participants in the food group consumed a standardized breakfast at 8:00 AM (determined by the individual’s BMI and containing 30% of the overall daily caloric needs (~ 450 kcal)^28^). At 10:00 AM, the participants in both groups underwent fear conditioning and ate a standardized lunch at 12:00 PM. All of the participants reported subjective hunger on a Visual Analog Scale (VAS) and were measured fingertip blood glucose levels after finishing the digit span test on day 1 and before fear memory training on day 2. The fear memory test occurred 24 h after fear training.

In Experiment 2, we investigated the effects of fasting on extinction and the return of fear. Forty-five participants were recruited. On day 1, after eating breakfast, the participants arrived at the laboratory at 8:00 AM and were randomly assigned to one of two groups: food group (*n* = 21) and fasting group (*n* = 24). After collecting demographic and cognitive data and measuring VAS hunger scores and fingertip blood glucose levels, all of the participants underwent fear conditioning at 10:00 AM. The participants in the fasting group were then subjected to acute food deprivation for 16 h beginning at 6:00 PM on day 1. To ensure that the participants complied with the instructions, we asked them to remain in the laboratory until noon the next day. On day 2, the participants in the food group consumed a standardized breakfast at 8:00 AM. After measuring VAS hunger scores and fingertip blood glucose levels, venous blood samples were collected 30 min before extinction training. All of the participants then ate a standardized lunch at 12:00 PM. The spontaneous recovery and reinstatement tests occurred 24 h after the end of extinction.

In Experiment 3, the participants from Experiment 2 were invited to return to the laboratory to assess the long-term effects of fasting on the return of fear 6 months later. Thirty subjects (food group: *n* = 13; fasting group: *n* = 17) from Experiment 2 participated in the 6-month follow-up study and underwent the spontaneous recovery and reinstatement tests of fear memory.

In Experiment 4, the experimental procedure was same as in Experiment 2. After fear conditioning on day 1, the subjects in the food group (*n* = 22) and fasting group (*n* = 23) underwent extinction on day 16, and venous blood samples were collected 30 min before extinction training. The fasting group was subjected to acute food deprivation for 16 h before extinction learning. On day 17, all of the participants underwent the spontaneous recovery and reinstatement tests.

In Experiment 5, to explore the mechanism underlying the effects of fasting on extinction retention and the return of fear, we measured the concentrations of plasma cortisol and ghrelin in the subjects from Experiments 2 and 4 after fasting for 16 h.

### Psychophysiological stimulation and assessment

Electric shocks were delivered by a constant-current STM200 stimulator (BIOPAC Systems, Goleta, CA, USA). A stimulating electrode was attached to the right inner wrist. The stimulus presentation was controlled by a computer using E-Prime software (Psychology Software Tools, Sharpsburg, PA, USA). The fear response was assessed by the SCR, which was measured using a BIOPAC MP150 system and analyzed using AcqKnowledge software (BIOPAC Systems) and recorded through shielded Ag-AgCl electrodes attached to the second and third fingers of the left hand. SCR data were low-pass filtered and smoothed. The greatest base to peak change in SCR in a 0- to 6-s window after each CS onset was assessed. These values were then square-root transformed to normalize the distribution.

### Hormone measurements

Venous blood samples were taken from all of the participants to determine plasma cortisol and ghrelin levels. The blood samples were centrifuged at 3000 rotations per minute at 4 °C for 10 min. The plasma samples were stored at − 80 °C until analysis. Plasma cortisol levels were measured using an enzyme-linked immunosorbent assay (ELISA; Cusabio Biotech, Hubei, China; catalog no. CSB-E05111h). Plasma ghrelin levels were measured using ELISA (Phoenix Pharmaceuticals, Belmont, CA, USA; catalog no. EK-031-30). The intra- and inter-assay coefficients of variation were < 8 and 10% for cortisol and < 10 and 15% for ghrelin.

### Statistical analysis

All of the statistical analyses were performed using SPSS 20.0 software (SPSS, Chicago, IL, USA). The data are expressed as mean ± standard error of mean (SEM) and were analyzed using independent-sample *t* tests and mixed-design analysis of variance (ANOVA) with appropriate between- and within-subjects factors (see Results). Pearson correlation coefficients (*r*) were used to evaluate relationships between the variables. Values of *P* *<* 0.05 were considered statistically significant.

## Results

### Fasting had no effect on fear memory acquisition

**Fig. 1:**
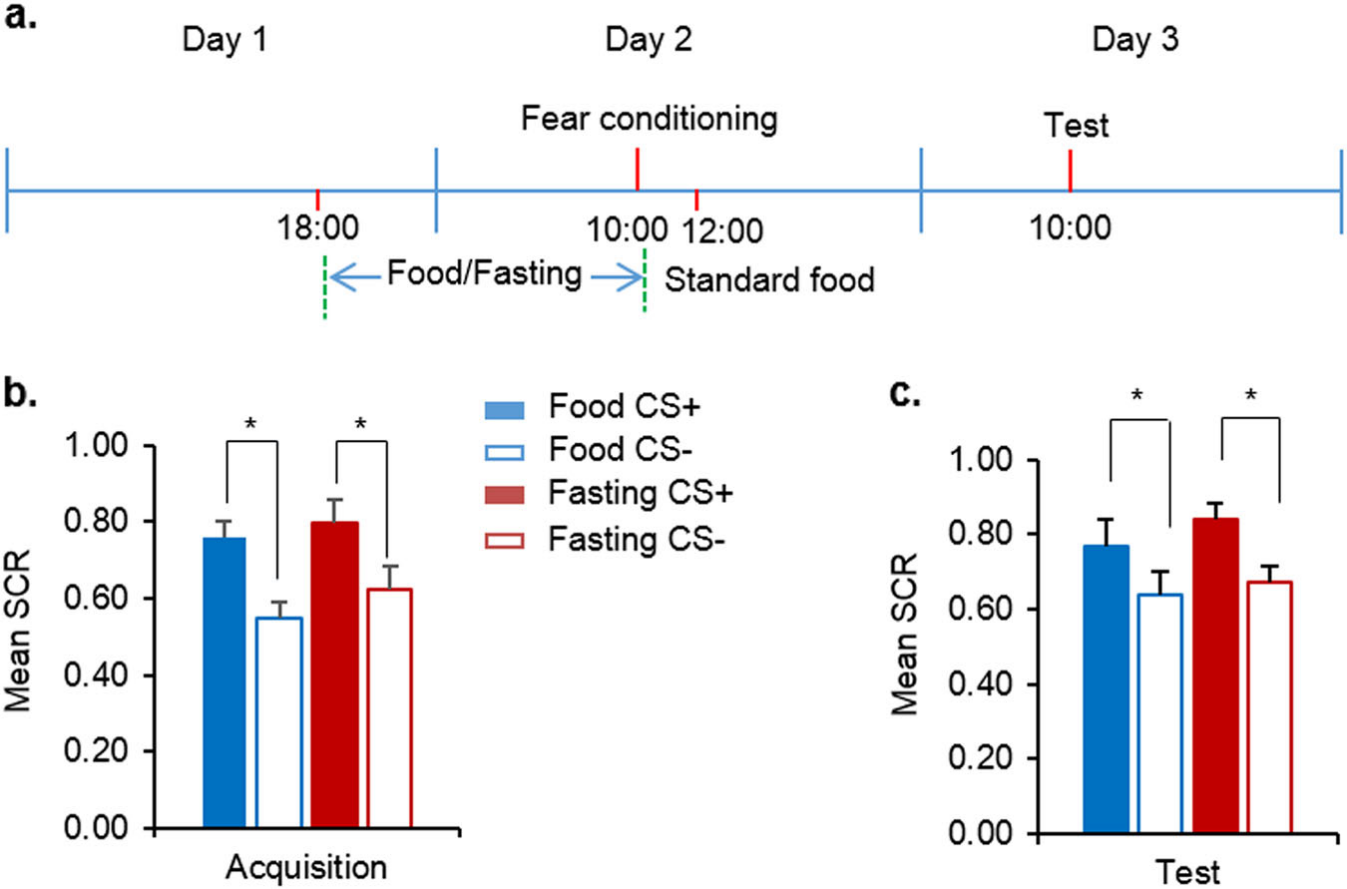
Effects of short-term fasting on fear acquisition. **a** Study procedure in Experiment 1. **b**, **c** Skin conductance response (SCR) during fear acquisition and the fear test. Food, *n* = 17; Fasting, *n* = 18. The data are expressed as mean ± SEM.*P *<* 0.05, compared with the SCR to the CS+ in the corresponding group

We first examined the effect of fasting on fear memory acquisition in Experiment 1 (Fig. 1a). No differences in age, education, height, weight, BMI, SDS, SAS, MoCA, digit span test (forward and backward), shock intensity, the subjective degree of hunger, or blood glucose levels on days 1 and 3 were found between the food group (*n* = 17) and fasting group (*n* = 18; all *P* *>* 0.05; Supplementary Table 1). The subjective degree of hunger and blood glucose levels on day 2 were significantly different between groups (*P* *<* 0.05; Supplementary Table 1). Fear conditioning was calculated using the average SCR during the all trials and the data was analyzed using mixed-design ANOVA, with group as the between-subjects factor (food and fasting) and CS (CS+ and CS−) as the within-subjects factor. This analysis revealed significant main effects of CS (F(1, 33) = 34.867, *P* *<* 0.001) but no main effect of group (F(1, 33) = 0.817, *P* = 0.373) and no CS × group interaction (F(1, 33) = 0.311, *P* = 0.581; Fig. 1b). Both groups showed greater SCR to CS+ compared with CS− during fear conditioning. This indicates that the subjects in both groups achieved successful and comparable acquisition of fear conditioning. During the fear memory test 24 h later, the mixed-design ANOVA revealed a significant main effect of CS (F(1, 33) = 15.697, *P* < 0.001) but no main effect of group (F(1, 33) = 1.103, *P* = 0.301) and no CS × group interaction (F(1, 33) = 0.070, *P* = 0.793; Fig. 1c). These results indicate that fasting had no effect on fear memory formation.

### Fasting enhanced extinction retention and prevented the return of fear

**Fig. 2:**
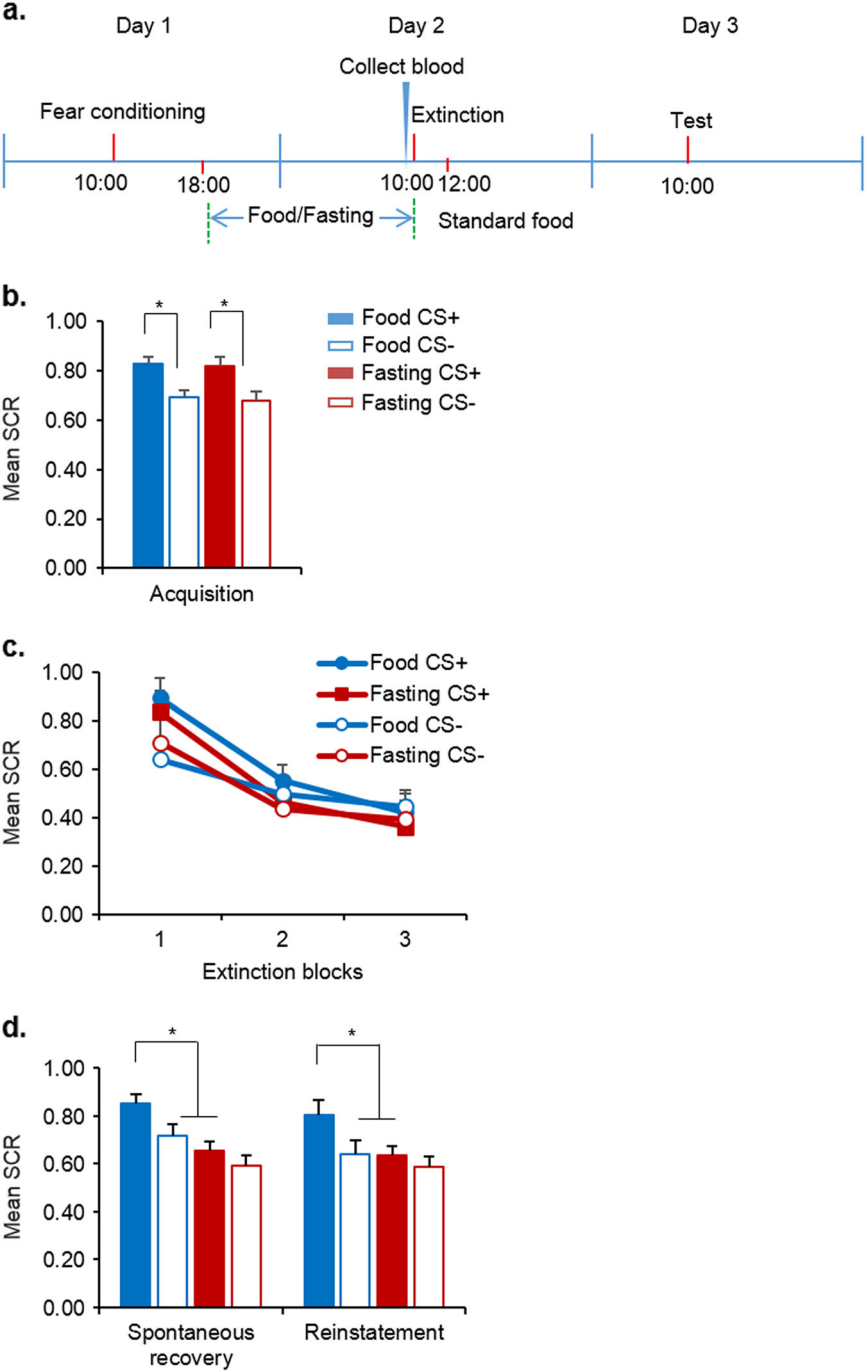
Effects of short-term fasting on fear extinction and fear return. **a** Study procedure in Experiment 2. **b** Skin conductance response (SCR) during acquisition. *P *<* 0.05, compared with the SCR to the CS+ in the corresponding group. **c** Skin conductance response during extinction. **d** Skin conductance response during the spontaneous recovery and reinstatement tests. *P *<* 0.05, compared with the SCR to the CS+ in the food group. The SCR to the CS+ in the fasting group was lower than that in the food group in the spontaneous recovery or reinstatement test. Food, *n* *=* 21; Fasting, *n* = 24. The data are expressed as mean ± SEM

In Experiment 2, we investigated whether fasting affected the extinction memory (Fig. 2a). No differences in age, education, height, weight, BMI, SDS, SAS, MoCA, digit span test (forward and backward), or shock intensity were found between the food group (*n* = 21) and fasting group (*n* = 24; all *P* *>* 0.05; Supplementary Table 2). During fear acquisition, the mixed-design ANOVA, with group (food and fasting) as the between-subjects factor and CS (CS+ and CS−) as the within-subjects factor, revealed main effect of CS (F(1, 43) = 129.419, *P* < 0.001) but no main effect of group (F(1, 43) = 0.059, *P* = 0.809) and no CS × group interaction (F(1, 43) = 0.046, *P* = 0.832; Fig. 2b). This showed that there were no differences in SCR during fear acquisition between participants assigned to the fasting and food groups.

To investigate the effect of fasting on fear extinction, the participants underwent extinction training 24 h after fear conditioning. The degree of hunger and blood glucose levels on day 2 were significantly different between groups (both *P* *<* 0.05; Supplementary Table 2). The SCR during extinction training was analyzed using mixed-design ANOVA, with extinction block (1–3) and CS (CS+ and CS−) as the within-subjects factors and group (food and fasting) as the between-subjects factor. We found that both groups displayed comparable extinction learning as evidenced by significant main effect of extinction block (F(2, 172) = 28.353, *P* < 0.001) but no main effects of group (F(1, 86) = 0.848, *P* = 0.360) and CS (F(1, 86) = 2.181, *P* = 0.143) and no extinction block × CS × group interaction (F(2, 172) = 0.191, *P* = 0.826; Fig. 2c). These results indicate that short-term fasting had no significant effect on extinction learning.

The spontaneous recovery test and reinstatement test were performed 24 h after fear extinction. The mixed-design ANOVA, with group (food and fasting) as the between-subjects factor and CS (CS+ and CS−) as the within-subjects factors, was used to analyze the SCR in the spontaneous recovery and reinstatement tests. The mixed-design ANOVA revealed significant main effects of group (F(1, 43) = 8.058, *P* = 0.007) and CS (F(1, 43) = 34.122, *P* < 0.001) and a significant group × CS interaction (F(1, 43) = 4.944, *P* = 0.031) in the spontaneous recovery test. In the reinstatement test, the ANOVA also revealed a significant main effect of CS (F(1, 43) = 20.134, *P* < 0.001) and a significant group × CS interaction (F(1, 43) = 5.735, *P* = 0.021). Follow-up *t* tests showed that the SCR to the CS− was comparable between groups in both the spontaneous recovery and reinstatement tests (both *P* *>* 0.05), and the SCR to the CS+ was higher in the food group than in the fasting group (both *P* *<* 0.05; Fig. 2d). These results indicate that 16 h of fasting enhanced extinction retention and prevented the return of fear.

### Fasting-induced enhancement of extinction retention was maintained for at least 6 months

**Fig. 3:**
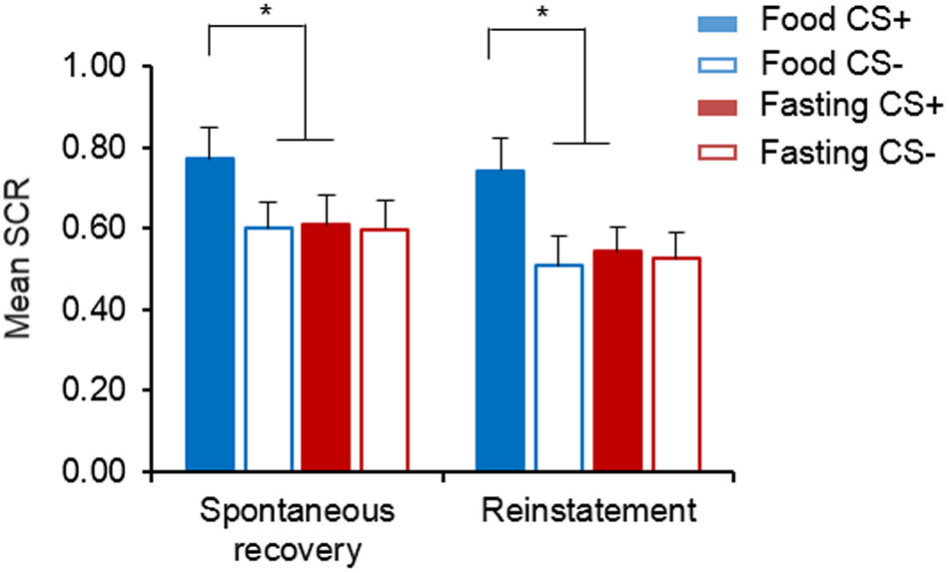
Persistence of fasting-induced enhancement of extinction retention. Skin conductance response (SCR) during the spontaneous recovery and reinstatement tests 6 months after extinction. *P *<* 0.05, compared with the SCR to the CS+ in the food group. The SCR to the CS+ in the fasting group was lower than that in the food group in the spontaneous recovery or reinstatement test. Food, *n* = 13; Fasting, *n* = 17. The data are expressed as mean ± SEM

In Experiment 3, 30 participants from Experiment 2 returned to the laboratory for the spontaneous recovery test and reinstatement test 6 months later. The mixed-design ANOVA revealed significant main effect of CS (F(1, 28) = 6.903 *P* = 0.014) and a significant group × CS interaction (F(1, 28) = 5.310, *P* = 0.029) in the spontaneous recovery test. In the reinstatement test, the mixed-design ANOVA also revealed a significant main effect of CS (F(1, 28) = 8.658, *P* = 0.006) and a significant group × CS interaction (F(1, 28) = 6.447, *P* = 0.017). Follow-up *t* tests showed that compared to the food group, the fasting group still had a significant lower conditioned fear to CS+ in the spontaneous recovery and reinstatement tests (both *P* *<* 0.05; Fig. 3). These results indicate that fasting led to better extinction retention and the long-lasting blockade of fear recovery.

### Extinction combined with fasting procedure inhibited remote fear memory

**Fig. 4:**
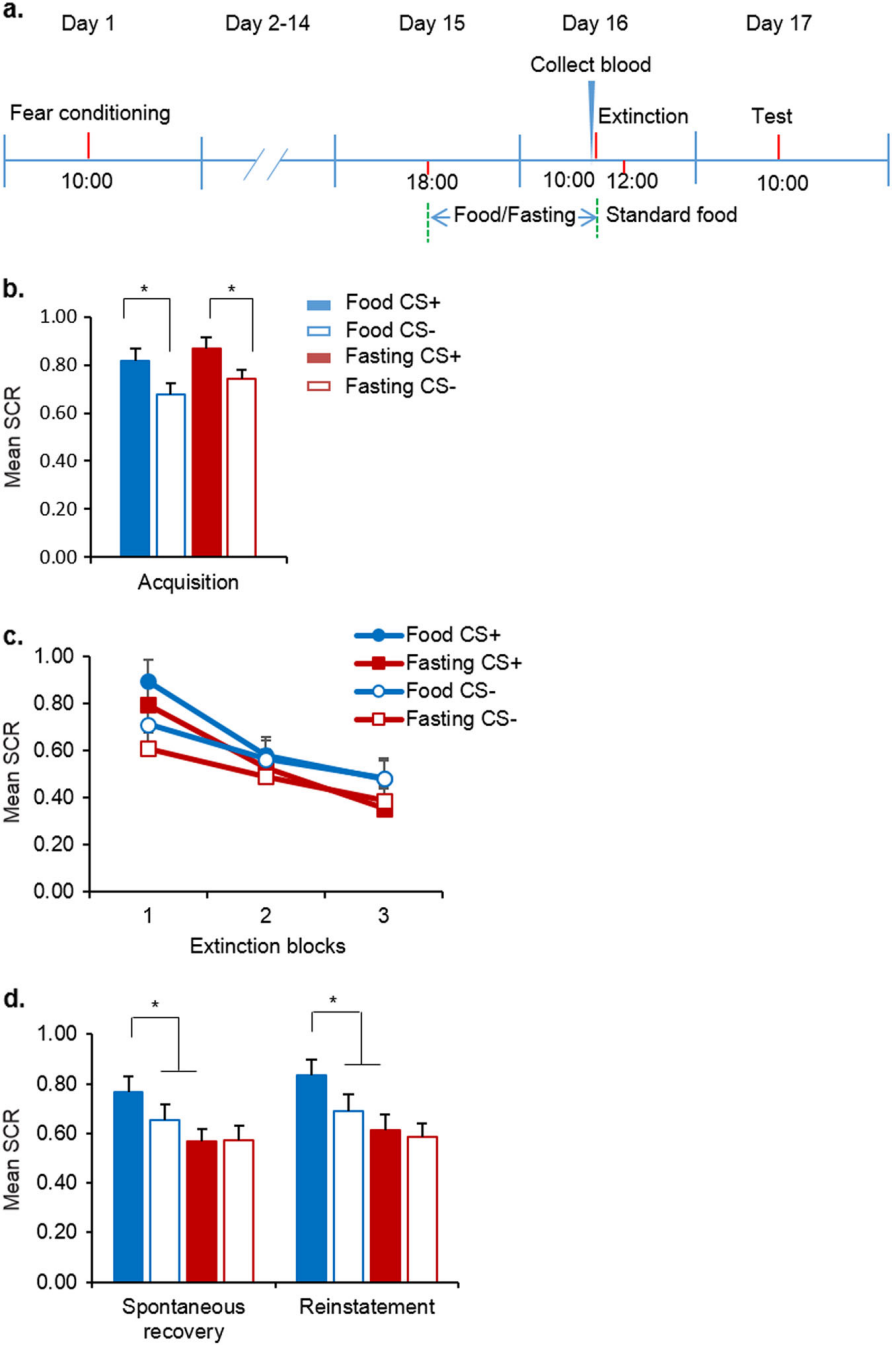
Effects of fasting on remote fear memory. **a** Study procedure in Experiment 4. **b** Skin conductance response (SCR) during acquisition. *P *<* 0.05, compared with the SCR to the CS+ in the corresponding group. **c** Skin conductance response during extinction. **d** Skin conductance response during the spontaneous recovery and reinstatement tests. *P *<* 0.05, compared with the SCR to the CS+ in the food group. The SCR to the CS+ in the fasting group was lower than that in the food group in the spontaneous recovery or reinstatement test. Food, *n* = 22; Fasting, *n* = 23. The data are expressed as mean ± SEM

In Experiment 4, we investigated whether the enhanced effect of fasting on extinction retention influences remote fear memory (Fig. 4a). No differences in age, education, height, weight, BMI, SDS, SAS, MoCA, digit span test (forward and backward), or shock intensity were found between groups (all *P* *>* 0.05; Supplementary Table 3). The mixed-design ANOVA of the SCR during fear conditioning, with group (food and fasting) as the between-subjects factor and CS (CS+ and CS−) as the within-subjects factor, revealed significant main effect of CS (F(1, 43) = 69.738, *P* < 0.001) but no main effect of group (F(1, 43) = 0.994, *P* = 0.324) and no CS × group interaction (F(1, 43) = 0.106, *P* = 0.746; Fig. 4b), indicating that both groups achieved successful and comparable acquisition.

Two weeks later, the participants returned to the laboratory. Before extinction began, the fasting group underwent 16 h of fasting. The degree of hunger and blood glucose levels on day 16 were significantly different between groups (both *P* *<* 0.05; Supplementary Table 3). The SCR during extinction training was analyzed using mixed-design ANOVA, with extinction block (1–3) and CS (CS+ and CS−) as the within-subjects factors and group (food and fasting) as the between-subjects factor. The analysis revealed main effect of extinction block (F(2, 172) = 35.791, *P* *<* 0.001) but no main effects of group (F(1, 86) = 2.487, *P* *=* 0.118) and CS (F(1, 86) = 1.219, *P* *=* 0.273) and no extinction block × group × CS interaction (F(2, 172) = 0.055, *P* = 0.947; Fig. 4c), indicating that different interventions did not show differential effects on fear responses to CS+ and CS− in extinction blocks.

The spontaneous recovery test and reinstatement test were performed 24 h after fear extinction. The mixed-design ANOVA, with group (food and fasting) as the between-subjects factor and CS (CS+ and CS−) as the within-subjects factor, revealed significant main effects of CS (spontaneous recovery: F(1, 43) = 4.972, *P* *=* 0.031; reinstatement: F(1, 43) = 12.579, *P* *=* 0.001), significant CS × group interactions (spontaneous recovery: F(1, 43) = 5.253, *P* *=* 0.027; reinstatement: F(1, 43) = 4.230, *P* *=* 0.046), and a main effect of group in the reinstatement test (F(1, 43) = 4.442, *P* *=* 0.041) but not in the spontaneous recovery test (F(1, 43) = 2.940, *P* *=* 0.094). Follow-up *t* tests showed that the SCR to the CS− in both tests was lower than the SCR to the CS+ in the food group, and the fear response to the CS+ in the fasting group was significantly lower than that in the food group (all *P* < 0.05; Fig. 4d). These results indicate that 16 h of fasting enhanced extinction retention and prevented the return of remote fear memory.

### Plasma ghrelin levels were associated with fasting-induced enhancement of extinction retention

**Fig. 5:**
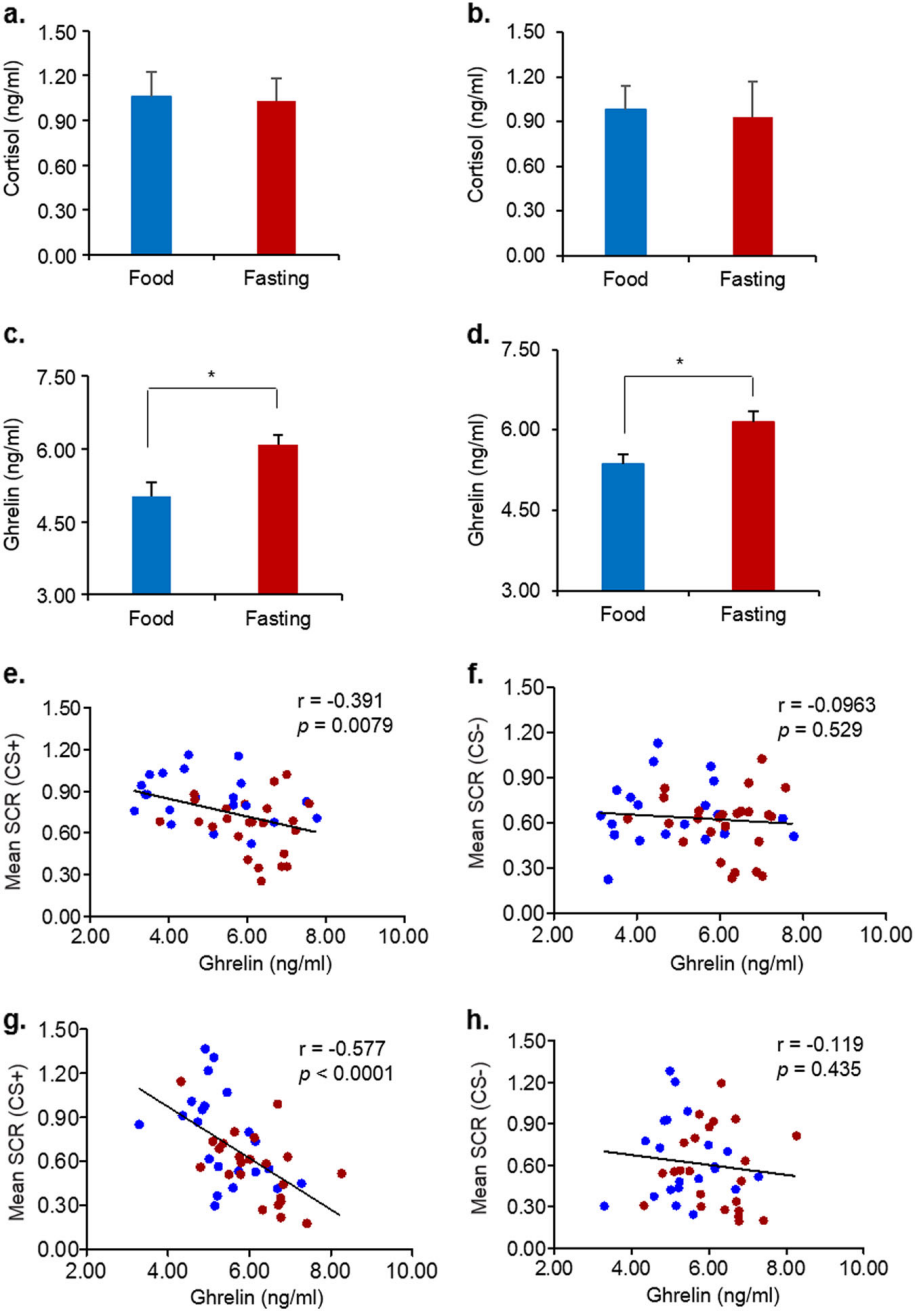
Effects of fasting on plasma cortisol and ghrelin levels. **a** Cortisol levels in Experiment 2. Food, *n* = 21; Fasting, *n* = 24. **b** Cortisol levels in Experiment 4. Food, *n* = 22; Fasting, *n* = 23. **c** Ghrelin levels in Experiment 2. *P *<* 0.05, compared with the food group. Food, *n* = 21; Fasting, *n* = 24. **d** Ghrelin levels in Experiment 4. *P *<* 0.05, compared with the food group. Food, *n* = 22; Fasting, *n* = 23. The data are expressed as mean ± SEM. **e** Pearson’s correlations between ghrelin and the fear response to CS+ in the spontaneous recovery test in Experiment 2. Food, *n* = 21; Fasting, *n* = 24. **f** Pearson’s correlations between ghrelin and the fear response to CS− in the spontaneous recovery test in Experiment 2. Food, *n* = 21; Fasting, *n* = 24. **g** Pearson’s correlations between ghrelin and the fear response to CS+ in the spontaneous recovery test in Experiment 4. Food, *n* = 22; Fasting, *n* = 23. **h** Pearson’s correlations between ghrelin and the fear response to CS− in the spontaneous recovery test in Experiment 4. Food, *n* = 22; Fasting, *n* = 23. Blue dots and red dots represent individuals in the food group and fasting group, respectively

No difference in cortisol levels was found between groups in Experiments 2 and 4 (both *P* *>* 0.05; Fig. 5a, b), indicating that the fasting protocol did not induce a lasting stress response. In the present study, we found that plasma ghrelin levels were increased after fasting in Experiments 2 (*t* (43) = − 3.070, *P* = 0.004; Fig. 5c) and 4 (*t* (43) = − 2.974, *P* = 0.005; Fig. 5d). Our analyses revealed a significant negative correlation between plasma ghrelin levels and SCR to the CS+ in the spontaneous recovery test (Experiment 2: Pearson *r* = −0.391, *P* = 0.0079, Fig. 5e; Experiment 4: Pearson *r* = − 0.577, *P* < 0.0001, Fig. 5g). There is no correlation between plasma ghrelin levels and SCR to the CS− in the spontaneous recovery test (Experiment 2: Pearson *r* = -0.0963, *P* = 0.529, Fig. 5f; Experiment 4: Pearson *r* = − 0.119, *P* = 0.435, Fig. 5h). These results indicate that the fasting-induced increase in ghrelin levels may be related to the decrease in fear response in the spontaneous recovery test.

## Discussion

In the present study, we tested a procedure to promote extinction retention and prevent the return of fear in humans. We evaluated the effects of short-term fasting on fear acquisition, extinction, and the return of fear. Our results demonstrated that fasting enhanced extinction retention and inhibited the subsequent return of fear in humans, with no effect on fear memory acquisition. Moreover, 16 h of fasting before extinction training effectively inhibited both recent and remote fear memory. We also found that the fasting-induced enhancement of extinction retention led to long-lasting blockade of the return of fear. Plasma ghrelin levels were elevated after fasting and had a negative relationship with the fear response at test. These findings provide evidence that fear extinction training combined with fasting may be useful for the treatment of fear-related disorders, with long-lasting effects.

Two studies reported that 16-h fasting promoted fear extinction in rodents^21,22^. The duration of fasting in human studies typically ranges from 2 to 24 h^24,29,30^. Overnight fasting for 12–14 h has been commonly used to detect relationships between fasting and cognition in humans^31,32^. Imaging findings showed that 14 or 16 h of overnight fasting significantly increased the activation of brain areas that are related to cognition^23,28^. Considering this, we imposed a fasting duration of 16 h in the present study. Further studies are required that extend the duration of fasting to cover the entire active phase of human activity.

Previous animal studies found that hunger has a significant impact on cognition and memory, such as increasing attention and promoting memory^33,34^. Fasting disrupts the latent inhibition of auditory fear conditioning in rats and facilitates aversive long-term memory formation in *Drosophila* and rodents^35,36^, and acute fasting promotes extinction training in rodents^21,22^. The present study found that fasting did not influence fear acquisition and extinction training in humans. These contradictory results between human and animal studies appear to be attributable to the different species and methods. Furthermore, a systematic review of the impact of fasting on cognition and memory in humans reported an inconsistent and incomplete profile of the effects of fasting on cognition^24^. This indicates the complexity of the effects of fasting on cognition and memory in humans. Differences in cognitive dimensions, memory tasks, ages, genders, and fasting durations may contribute to these inconsistent results.

In our study, we found that fasting enhanced extinction retention and prevented fear return. The fear response was shown to extinguish after repeated exposure to the CS without the US during extinction^37^. Extinction is not the same as forgetting, and the decrease in the conditioned fear response after CS–no-US memory training is generally not permanent. There are several instances in which extinguished fear responses reappear, such as with spontaneous recovery and reinstatement^6,8,38^. The present study found no spontaneous recovery or reinstatement after extinction after overnight fasting. These findings suggest that extinction retention after fasting conditions was more stable, more persistent, and resistant to spontaneous recovery and reinstatement. There may be several explanations for these findings.

First, fasting as a physiological state affects the transport of appetite-related hormones, such as ghrelin and orexin, into the brain^39,40^. Such hormones play important roles in regulating synaptic plasticity^41–44^. Fasting was shown to elevate excitatory synaptic inputs through the orexigenic hormone ghrelin^45^. Ghrelin mediates the facilitatory effect of fasting on fear extinction and its retention in mice and mimics the effect of fasting on the impairments of paired-pulse low-frequency stimulation and long-term depression^21^. In the present study, we found that plasma ghrelin levels were elevated after fasting, and a significant negative correlation was found between ghrelin and the fear response in the spontaneous recovery test. Previous studies showed that ghrelin is secreted in a pulsatile pattern and is not influenced by the time of day. Nonetheless, a characteristic diurnal course with spontaneous rises and declines at customary mealtimes has been reported^46,47^. In the present study, to control for ghrelin secretion that is influenced by food intake and circadian rhythm, all of the subjects were enrolled with regular lifestyles and habitual dieting, and they experienced food deprivation or intake at the same time. However, the patterns of baseline ghrelin release might have been different across subjects, thus causing possible bias. Short-term fasting also may induce the synchronous release of several neuromodulators and hormones that ultimately promote synaptic plasticity in the process of extinction. Fasting-induced synaptic plasticity may allow fear erasure through the extinction-guided remodeling of memory circuitry. Orexins are secreted by the hypothalamus and regulate feeding behavior^48^. In rodents, orexin receptor-1 blockade in the amygdala was beneficial for the consolidation of extinction memory^49^. However, our previous study found that plasma orexin levels in humans before extinction were negatively associated with performance on recent fear memory tests but not remote fear memory tests^50^. These results indicate that the orexin system may play different roles in fear extinction across species. Neuropeptide Y and pancreatic polypeptide promote fear extinction by acting on Y_4_ receptors in the central nervous system^51^. Another gut-brain-related peptide, glucagon-like peptide-1, can bind the glucagon-like peptide-1 receptor in the hippocampus to enhance memory^52^. However, the role of glucagon-like peptide-1 in fear extinction is unknown. Brain-derived neurotrophic factor is a widely studied neurotrophin that is closely related to feeding behavior, learning, and memory^53^. Numerous studies have found that brain-derived neurotrophic factor is a key regulator that improves fear extinction^54–56^. Further confirmation of whether the fasting-induced enhancement of extinction retention is related to neural plasticity changes caused by these peptides or neurotrophins is needed.

Second, glucose has been shown to influence memory. Glucose metabolism provides the energy for physiological brain function through the generation of adenosine triphosphate. The memory-improving effect of glucose has been studied for many years in both animals and humans^57,58^. However, a previous study found that higher blood glucose levels had a negative influence on cognition, possibly mediated by impairments in hippocampal microstructure^59^. Strategies that seek to lower glucose levels may beneficially influence cognition and memory. One possibility is that low blood glucose levels that are caused by fasting may have memory retention-enhancing effects.

Third, fasting can induce changes in emotion-related brain activity. Activation of the amygdala, orbitofrontal cortex, and hippocampus was shown to be involved in the hunger-induced enhancement of memory in humans^17,18^. Previous studies reported that three interconnected brain regions are involved in fear memory extinction: amygdala, prefrontal cortex, and hippocampus^19^. A recent study found that hunger promoted fear extinction consolidation by activating microcircuitry in the amygdala^22^. Altogether, the enhancement of extinction memory maintenance may be attributable to fasting-induced activation of the amygdala, orbitofrontal cortex, and hippocampus, thus contributing to extinction memory consolidation and inhibition of the return of the original fear memory. Further neuroimaging studies are needed to clarify the involvement of specific brain regions.

Previous studies showed that cortisol reduces fear recall and facilitates the consolidation of extinction memory in both animals and humans^60,61^. We also investigated whether fasting induces a stress response that influences extinction memory. Fasting did not increase plasma cortisol levels, demonstrating that the fasting-induced enhancement of extinction memory retention was not attributable to a fasting-induced stress response. A previous study found that 2-day fasting evoked moderate stress but did not affect mood, brain activity, or cognition^62^. One possibility is that 16 h of fasting is insufficient to induce a stress response.

The generalizability of our findings is limited because we recruited only male volunteers. Considering that hormone levels are different between men and women^63^, we did not include female subjects. We also recognize that sample size in each group was limited. We plan to extend this procedure to participants of both sexes and different ages using large sample sizes in future studies. Another potential issue is that Pavlovian fear conditioning was performed in a laboratory setting that cannot entirely recapitulate the clinical characteristics of fear-related disorders. Moreover, although the Individuals in both the fasting group and food group ate during extinction memory consolidation, we cannot exclude the possible effect of eating after fasting on fear extinction retention. Future studies should test whether fasting combined with extinction-based therapy can decrease the fear response and anxiety in patients.

In conclusion, the present study tested a new behavioral procedure (short-term fasting combined with extinction) to eliminate and inhibit fear responses. Beneficial effects of this procedure persisted for at least 6 months. This procedure may have significant value for clinical applications. Future studies should determine the neural mechanisms that are involved in the effects of this procedure and extend the procedure to clinical populations.

## Electronic supplementary material

Supplemental table
