# Supplementary material for: Fasting enhances extinction retention and prevents the return of fear in humans
Source: Transl Psychiatry. 2018 Oct 9;8:214. doi: 10.1038/s41398-018-0260-1 (PMC6177454; doi:10.1038/s41398-018-0260-1)
Supplement: Supplementary file 1 — Supplemental table [file 41398_2018_260_MOESM1_ESM.docx]

Supplementary Table 1. Demographic data, shock intensity, and degree of hunger in Experiment 1.

| **Group** | | **Food** | **Fasting** | ***P*** |
| --- | --- | --- | --- | --- |
| Age (years) | | 24.12 ± 0.61 | 25.17 ± 0.83 | 0.32 |
| Education (years) | | 16.82 ± 0.57 | 16.72 ± 0.50 | 0.89 |
| Height (cm) | | 174.24 ± 1.71 | 174.00 ± 1.21 | 0.91 |
| Weight (kg) | | 70.41 ± 2.73 | 71.72 ± 2.49 | 0.72 |
| BMI | | 23.06 ± 0.50 | 23.62 ± 0.63 | 0.50 |
| SDS | score | 28.53 ± 1.43 | 29.78 ± 1.95 | 0.61 |
|  | standard score | 35.66 ± 1.79 | 37.22 ± 2.44 | 0.61 |
| SAS | score | 26.71 ± 1.64 | 26.17 ± 1.27 | 0.79 |
|  | standard score | 33.38 ± 2.05 | 32.71 ± 1.59 | 0.79 |
| MoCA | | 28.00 ± 0.24 | 27.89 ± 0.40 | 0.81 |
| Digit span test | forward | 9.35 ± 0.32 | 9.39 ± 0.33 | 0.94 |
|  | backward | 7.06 ± 0.31 | 6.72 ± 0.33 | 0.49 |
| Shock intensity | | 41.45 ± 1.94 | 42.52 ± 2.56 | 0.74 |
| Glucose (mmol/L) | day 1 | 6.63 ± 0.24 | 5.94 ± 0.25 | 0.06 |
|  | day 2 | 6.33 ± 0.21 | 4.92 ± 0.13* | 0.00* |
| Degree of hunger | day 1 | 1.87 ± 0.44 | 1.25 ± 0.43 | 0.30 |
|  | day 2 | 2.53 ± 0.46 | 7.15 ± 0.35* | 0.00* |
|  | day 3 | 1.39 ± 0.35 | 0.79 ± 0.19 | 0.13 |

The results are expressed as mean ± SEM. BMI, Body Mass Index; SDS, Self-rating Depression Scale; SAS, Self-rating Anxiety Scale; MoCA, Montreal Cognitive Assessment. **P* < 0.05, compared with food group.

Supplementary Table 2. Demographic data, shock intensity, and degree of hunger in Experiment 2.

| **Group** | | **Food** | **Fasting** | ***P*** |
| --- | --- | --- | --- | --- |
| Age (years) | | 24.05 ± 0.47 | 23.33 ± 0.39 | 0.10 |
| Education (years) | | 16.95 ± 0.42 | 16.04 ± 0.34 | 0.74 |
| Height (cm) | | 175.24 ± 1.16 | 174.75 ± 0.91 | 0.09 |
| Weight (kg) | | 70.33 ± 1.51 | 66.50 ± 1.55 | 0.09 |
| BMI | | 22.90 ± 0.43 | 21.77 ± 0.47 | 0.12 |
| SDS | score | 30.38 ± 0.96 | 28.42 ± 0.81 | 0.09 |
|  | standard score | 37.67 ± 1.18 | 34.75 ± 1.16 | 0.46 |
| SAS | score | 27.14 ± 1.03 | 28.17 ± 0.93 | 0.45 |
|  | standard score | 33.57 ± 1.30 | 34.92 ± 1.18 | 0.92 |
| MoCA | | 28.00 ± 0.32 | 27.96 ± 0.27 | 0.84 |
| Digit span test | forward | 9.00 ± 0.29 | 9.08 ± 0.28 | 0.21 |
|  | backward | 6.67 ± 0.31 | 7.21 ± 0.29 | 0.49 |
| Shock intensity | | 46.17 ± 2.21 | 48.95 ± 3.15 | 0.49 |
| Glucose (mmol/L) | day 1 | 6.16 ± 0.19 | 6.37 ± 0.16 | 0.60 |
|  | day 2 | 6.12 ± 0.15 | 5.27 ± 0.12* | 0.00* |
| Degree of hunger | day 1 | 1.33 ± 0.35 | 1.35 ± 0.41 | 0.98 |
|  | day 2 | 2.03 ± 0.36 | 7.50 ± 0.32* | 0.00* |
|  | day 3 | 1.01 ± 0.38 | 1.63 ± 0.35 | 0.24 |

The results are expressed as mean ± SEM. BMI, Body Mass Index; SDS, Self-rating Depression Scale; SAS, Self-rating Anxiety Scale; MoCA, Montreal Cognitive Assessment. **P* < 0.05, compared with food group.

Supplementary Table 3. Demographic data, shock intensity, and degree of hunger in Experiment 4.

| **Group** | | **Food** | **Fasting** | ***P*** |
| --- | --- | --- | --- | --- |
| Age (years) | | 23.82 ± 0.54 | 24.52 ± 0.38 | 0.29 |
| Education (years) | | 17.23 ± 0.43 | 17.52 ± 0.3 | 0.58 |
| Height (cm) | | 173.36 ± 0.93 | 174.00 ± 1.21 | 0.68 |
| Weight (kg) | | 65.50 ± 2.60 | 70.22 ± 1.98 | 0.15 |
| BMI | | 21.74 ± 0.78 | 23.14 ± 0.52 | 0.14 |
| SDS | score | 27.64 ± 1.22 | 28.26 ± 1.13 | 0.71 |
|  | standard score | 34.27 ± 1.54 | 34.91 ± 1.42 | 0.76 |
| SAS | score | 28.00 ± 1.28 | 26.91 ± 0.96 | 0.50 |
|  | standard score | 34.73 ± 1.59 | 33.43 ± 1.21 | 0.52 |
| MoCA | | 27.95 ± 0.28 | 27.87 ± 0.37 | 0.86 |
| Digit span test | forward | 9.68 ± 0.27 | 9.61 ± 0.30 | 0.86 |
|  | backward | 8.27 ± 0.34 | 8.00 ± 0.26 | 0.53 |
| Shock intensity | | 50.77 ± 2.41 | 47.53 ± 1.98 | 0.30 |
| Glucose (mmol/L) | day 1 | 6.05 ± 0.24 | 6.33 ± 0.16 | 0.33 |
|  | day 2 | 5.98 ± 0.22 | 4.71 ± 0.15* | 0.00* |
| Degree of hunger | day 1 | 1.47 ± 0.38 | 1.13 ± 0.30 | 0.48 |
|  | day 2 | 2.22 ± 0.51 | 8.00 ± 0.36* | 0.00* |
|  | day 3 | 1.56 ± 0.48 | 1.16 ± 0.27 | 0.45 |

The results are expressed as mean ± SEM. BMI, Body Mass Index; SDS, Self-rating Depression Scale; SAS, Self-rating Anxiety Scale; MOCA, Montreal Cognitive Assessment. **P* < 0.05, compared with food group.
